# Deep Soil C, N, and P Stocks and Stoichiometry in Response to Land Use Patterns in the Loess Hilly Region of China

**DOI:** 10.1371/journal.pone.0159075

**Published:** 2016-07-14

**Authors:** Changzhen Li, Luhong Zhao, Pingsheng Sun, Fazhu Zhao, Di Kang, Gaihe Yang, Xinhui Han, Yongzhong Feng, Guangxin Ren

**Affiliations:** 1 College of Agronomy, Northwest A&F University, Yangling, Shaanxi, China; 2 The Research Center of Recycle Agricultural Engineering and Technology of Shaanxi Province, Yangling, Shaanxi, China; 3 College of Forestry, Northwest A&F University, Yangling, Shaanxi, China; 4 College of Urban and Environmental Sciences, Northwest University, Xi’an, Shaanxi, China; Tennessee State University, UNITED STATES

## Abstract

In the Loess Hilly Region of China, the widespread conversion of cropland to forestland and grassland has resulted in great increased in organic carbon (C), nitrogen (N) and phosphorus (P) stocks in the shallow soil layers. However, knowledge regarding changes in C, N, and P in deep soil is still limited. To elucidate the responses of deep soil C, N, and P stocks and stoichiometry in response to changes in land use, the soil from a 0–200 cm soil profile was collected from the following three typical land use patterns in the heartland of the region: forestland, grassland, and cropland. Compared with cropland, forestland and grassland had improved soil organic carbon (SOC) and total nitrogen (TN) contents and stocks at most soil depths but decreased total phosphorus (TP) contents and stocks. At soil depths of 0–200 cm in the forestland and grassland, the cumulative SOC stocks were improved by 34.97% and 7.61%, respectively, and the TN stocks were improved by 54.54% and 12.47%, respectively. The forestland had higher SOC, TN and TP contents and stocks compared to the grassland in almost all soil layers. The soil depths of 100–200 cm contained the highest percentages of SOC, TN and TP stocks (47.80%–49.93%, 46.08%–50.05% and 49.09%–52.98%, respectively). Additionally, the forestland and grassland showed enhanced soil C:P, N:P and C:N:P ratios, and the forestland had higher C:P, N:P and C:N:P ratios compared to the grassland. Furthermore, the SOC and TN stocks had significant impacts on the soil C:N, C:P and N:P ratios. It was concluded that afforestation was the best choice for soil nutrient restoration of degraded land, and deep soil provided an extremely important resource for evaluating soil C, N and P pools and cycling.

## Introduction

Soil plays a significant role in the global carbon cycle [1] and stores approximately 2500 Pg of carbon (C) globally, which is 3.3 and 4.5 times the size of the atmospheric and biotic C pools, respectively [2]. Thus, soil can act either as a C source or as a C sink for atmospheric CO_2_ [3] and has been considered a key research area in the scientiﬁc debate on global climate change [4]. Land use changes disturb the equilibrium between C inflows and outflows in the soil [5], causing the perturbation of the ecosystem and inﬂuencing the C stocks [6]. Globally, 22% and 59% of the soil C stock has been lost through the conversion of forests and pastures, respectively, to cropland [5, 7]. When land use changes decrease the soil C, the reverse process usually increases the soil C [5]. After land use is changed from agricultural land to forest, fallow, and grassland, the soil C stocks have been shown to increase by 29%, 32% and 26%, respectively [8]. Vegetation reconstruction is likely to affect the C cycle and the amount of C stored in the soil due to changes in the vegetation biomass, decomposition, erosion, and turnover rates [9]. Soil nitrogen (N) and phosphorus (P) cycling is tightly coupled to C cycling in ecosystems, and can also be altered by revegetation and related management practices [10]. Previous studies had reported the effects of land use changes on soils at different spatial scales, including the local [11–13], the regional [14, 15], the national [9, 16], and the global [8], but most of them primarily focused on topsoil. Rumpel and Kögel-Knabner [17] reported that subsoil had a high potential to sequester CO_2_ from the atmosphere, and more than 50% of total soil C was stored in the subsoil. Shi et al. [18] showed that soil organic carbon (SOC) stored in the top meter of soil (20–100 cm) ranged between 50% and 67%. A recent study focusing on SOC pool of 0–300 cm soil layer in the northern circumpolar permafrost region, suggested that at least 81% of soil C was stored below 30 cm, approximately 52% was stored in 100–300 cm [19]. Thus, deep soil may play an even more important role as CO_2_ sink than topsoil. Soil cannot store unlimited amount of C and the saturation threshold exists [11, 20]. Understanding the C stock in deep soil is essential to ascertain soil C threshold and explore ecosystem C cycle. Gao et al. [21] reported that ecological restoration must start with the restoration of soil fertility. Estimating soil C, N, and P stocks induced by land use change is also necessary to research soil nutrient threshold under different vegetation and develop restoration strategies for degraded ecosystems. However, the revegetation-induced changes in the C, N and P statuses in deep soil are still poorly understood.

In general, C, N and P are present in relatively stable ratios in living organisms [22]. Land use changes and related management practices have a signiﬁcant impact on soil C, N, and P stoichiometry [23–28]. Elements and organisms in environments interact with each other by absorbing or releasing different elements, and elemental ratios are similarly regulated [22, 29]. Vegetation cover, plant types and communities, geomorphology, and seawalls all affect the stoichiometry of C, N, and P in soils [30]. Furthermore, the key characteristics of organisms and ecosystems are determined by the dynamics of element ratios [31]. Globally, the C:N:P ratios of 186:13:1 and 60:7:1 have been determined to be well-balanced in soil and soil organisms, respectively [13, 32]. Soil C:N:P ratios could be good indicators of soil nutrient status in organic-rich topsoil [33]. Thus, exploring the soil stoichiometry of C, N and P in terrestrial ecosystems is of significant importance to nutrient supplies in plants [22]. However, the stoichiometry of C, N and P is not very clear in the soil, especially in the subsoil or even in deep soil [34].

The Loess Hilly Region of China, which has an area of 62.4 × 10^4^ km^2^, is considered one of the most seriously eroded areas in the world [14, 15, 35] and plays an important role in global C cycle [36]. The average erosion rate is 150 Mg/ha per year before the Chinese government management [37] and the total erosion area has reached 72.3% [12]. Soil erosion and desertiﬁcation have caused a loss of net primary productivity as high as 12 kg C/ha per year [38]. To control soil erosion and restore ecosystems, the Chinese government has undertaken many efforts since the 1950s [4, 34]. One of the most ambitious ecological reconstruction programs is the "Grain for Green" program, which was the first "payment-for-ecosystem-services" program in China implemented in 1999 [9, 14]. The aim of the program was to convert approximately 2.04 × 10^6^ ha of low-yield croplands with slopes greater than 15° into forestland and grassland in the Loess Hilly Region [14]. Although the initial goal of the program was to control soil erosion, it also changed the C, N and P stocks in the terrestrial ecosystems. However, less is known about the dynamics, stocks and stoichiometry of C, N and P in the deep soil after long-term revegetation in this region. Thus, the objectives of this study were to investigate: (1) the distribution patterns of soil C, N and P contents in the soil profile induced by revegetation; (2) the effects of land use types and soil depths on C, N, P stocks and stoichiometry; and (3) the factors affecting the soil C:N, C:P and N:P ratios.

## Materials and Methods

The history of the sites was determined through interviews with local forest ranger (Mr. Xueqing Zhao, Zhenwudong Town, Ansai County, Shaanxi, China). The land accessed is not privately owned or protected in any way and the field studies did not involve endangered or protected species.

### Study area

This study was conducted in the Wuliwan catchment (36°46′42″-36°46′″28″N, 109°13′46″-109°16′03″E), which is located in Ansai County at the central region of the hilly Loess Plateau, China. The study area was characterized by a semi-arid climate and a hilly-gully Loess landscape. The recent annual average temperature is 9.1°C. The highest temperature is 35.3°C in summer and the lowest temperature is -20.3°C in winter. The annual average precipitation is 503 mm, 70% of which occurs between July and September. On average, the frost-free period is 157 days and the sunshine duration is 2415 h every year. The accumulated temperatures above 0°C and 10°C are 3733°C and 3283 °C, respectively. Most of the arable cropland occupies slope lands without irrigation and with slopes varying between 0° and 65°. The loess-derived soil is highly erodible.

The major agricultural land use pattern in the study area is slopeland, and maize (*Zea mays L*.) is the major crop. Historically, the native vegetation was destroyed due to the needs of food from an expanding population, which resulted in severe soil erosion and land degradation [39]. Since the 1950s, considerable work has been done to control soil erosion and restore vegetation [40], mainly including extensive reforestation of the Loess Plateau in the 1970s and the integrated soil erosion control at the watershed scale in the 1980s and 1990s [41]. Agricultural management has not been changed significantly in this region since the 1970s. At the study site, *Robinia pseudoacacia* L. is the dominant tree in the artificial forest. During the afforestation period, natural grassland was also generated from abandoned cropland due to the low productivity and long distances from farmers’ residences [34]. Wild grasslands and shrub lands were also found on steep slopes for firewood collection, resulting in limited vegetation coverage or even barren for long periods. In 1999, most slopelands were closed for vegetation restoration under the “Grain for Green” program [14].

### Experimental design

The conversion of cropland into artificial forestland (20-year *Robinia pseudoacacia*) and natural grassland (20-year abandoned cropland) at the Wuliwan catchment was investigated to assess the effects of land use types on soil C, N, P stocks and stoichiometry. Maize cropland near the site with low levels of fertilizer and no irrigation was selected for comparison. In June 2015, we randomly selected three lands for each land use type. Three 30 m × 30 m plots in each land were portioned for use as the experimental sites.

### Field investigation, soil sampling and analysis

Six quadrats were separately chosen along the diagonal in each plot. Quadrats of 10 m × 10 m were established for forestland to determine canopy closure, height and diameter at breast height (DBH). The average canopy closure, mean height and DBH of the artificial forest were 55%, 5.44 m and 9.23 cm, respectively. Quadrats of 1 m × 1 m were established for grassland to determine herb coverage and species. The average herb coverage was 65%, and the major species were *Astragalus melilotoides Pall*., *Artemisia sacrorum Ledeb*, and *Poa sphondylodes Trin*.

Soil samples were taken from ten points in an “S” shape from each plot. The soil was sampled using a stainless steel auger with a diameter of 5 cm with 10-cm depth intervals at depths of 0–100 cm and with 20-cm depth intervals at depths of 100–200 cm. The ground litter was removed before soil sampling. Then, the soil samples in each layer were collected at ten points and were mixed together to form one sample. The samples were collected at least 80 cm away from the trees. The leaves, roots and lager debris were removed, and all samples were sieved through a 2 mm screen. The samples were air-dried and stored at room temperature for the determination of the soil chemical properties. The soil bulk density (BD) samples were collected with a ring tube in each soil depth and dried in an oven at 104°C for 48 h.

The SOC, total nitrogen (TN) and total phosphorus (TP) contents were determined with the dichromate oxidation method, Kjeldahl procedure after digestion with concentrated H_2_SO_4_ on a distillation unit, and HClO_4_-H_2_SO_4_ ammonium molybdate ascorbic acid method, respectively [42].

### Calculations and statistical analysis

We used the following equation to calculate SOC (TN and TP) stocks (Cs (Ns and Ps)) [5]:

Cs=BD×SOC×D/10

where Cs is the SOC stock (Mg·ha^-1^), BD is the soil bulk density (g·cm^-3^), SOC is the soil organic carbon content (g·kg^-1^), and D is the soil sampling thickness (cm).

All statistical analysis was performed using the software program SAS, ver. 8.1. One-way analysis of variance (ANOVA) was used to analyze the means of the same soil layer among the different revegetation types. Two-way analysis of variance (ANOVA) was used to determine the effects of land use patterns, soil depth, and their interactions on the SOC, TN and TP contents, stocks, and stoichiometry. Regression analysis was used to test relationships between soil stock and stoichiometric characteristics in topsoil and deep soil. The differences were evaluated at the 5% significance level. When significance was observed at the P < 0.05 level, Duncan’s multiple Range Test was used to carry out the multiple comparisons. Pearson linear correlation coefficients analysis was used to estimate the relationships between the stocks and stoichiometry of SOC, TN and TP.

## Results

### Changes in C, N and P concents

**Fig 1:**
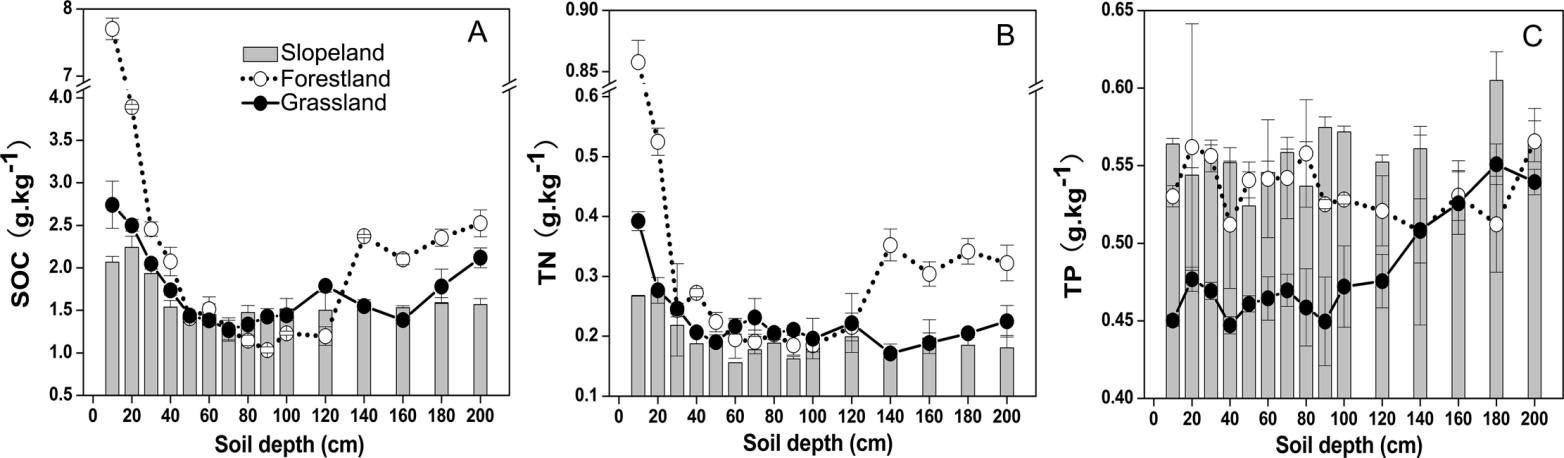
**Dynamic distribution of SOC (A), TN (B) and TP (C) concents at a soil depth of 0**–**200 cm.** The error bars are the standard errors.

The SOC, TN and TP contents were different with increasing soil depths (Fig 1). In all land use patterns, the highest SOC and TN contents were in the topsoil (0–10 cm), with 2.07–7.70 g·kg^-1^ and 0.27–0.86 g·kg^-1^, respectively (Fig 1A and 1B). The SOC, TN and TP contents decreased significantly at soil depths of 10–40 cm, while in the subsoil (40–100 cm), the changes tended to be slower (Fig 1A, 1B and 1C). In addition, the SOC, TN and TP contents increased in the deep subsoil (100–200 cm).

The land use patterns significantly altered the SOC, TN and TP contents (P < 0.05) (Fig 1). The SOC and TN contents of forestland were significantly higher than those of grassland and cropland at soil depths of 0–40 cm and 120–200 cm (Fig 1A and 1B). The TP content of forestland was greater than that of grassland at soil depths of 0–120 cm, but forestland and grassland both had lower TP contents than cropland at almost all soil depths (0–200 cm) (Fig 1C). At soil depths of 0–200 cm, the average SOC, TN and TP contents of forestland, grassland and cropland were as follows: 2.28 g·kg^-1^, 1.73 g·kg^-1^ and 1.61 g·kg^-1^, respectively (SOC); 0.31 g·kg^-1^, 0.23 g·kg^-1^ and 0.20 g·kg^-1^, respectively (TN); and 0.54 g·kg^-1^, 0.48 g·kg^-1^ and 0.56 g·kg^-1^, respectively (TP). A two-way ANOVA indicated that land use patterns and soil depth both significantly affected the SOC, TN and TP contents. Additionally, the interactions between land use patterns and soil depth were also significant (Table 1).

**Table 1:** *F* and *P* values for the effects of land use patterns and soil depth on the soil C:N:P stoichiometry in the hilly area of Loess Plateau, China. These three factors concern soil stocks or ratios.

Factor	*F* (*P*) value								
	C	N	P	Cs	Ns	Ps	C:N	C:P	N:P
**Land use patterns**	336.30 (< .0001)	287.49 (< .0001)	72.75 (< .0001)	216.03 (< .0001)	135.90 (< .0001)	51.58 (< .0001)	7.87 (0.0024)	375.68 (< .0001)	692.96 (< .0001)
**Soil depth**	303.31 (< .0001)	113.11 (< .0001)	1.97 (0.0288)	2776.01 (< .0001)	841.67 (< .0001)	5882.72 (< .0001)	11.61 (< .0001)	555.26 (< .0001)	875.46 (< .0001)
**Land use patterns × soil depth**	115.66 (< .0001)	37.22 (< .0001)	2.26 (0.0020)	51.53 (< .0001)	27.12 (< .0001)	10.11 (< .0001)	14.00 (< .0001)	229.16 (< .0001)	247.06 (< .0001)

### Changes in SOC, TN and TP stocks

**Fig 2:**
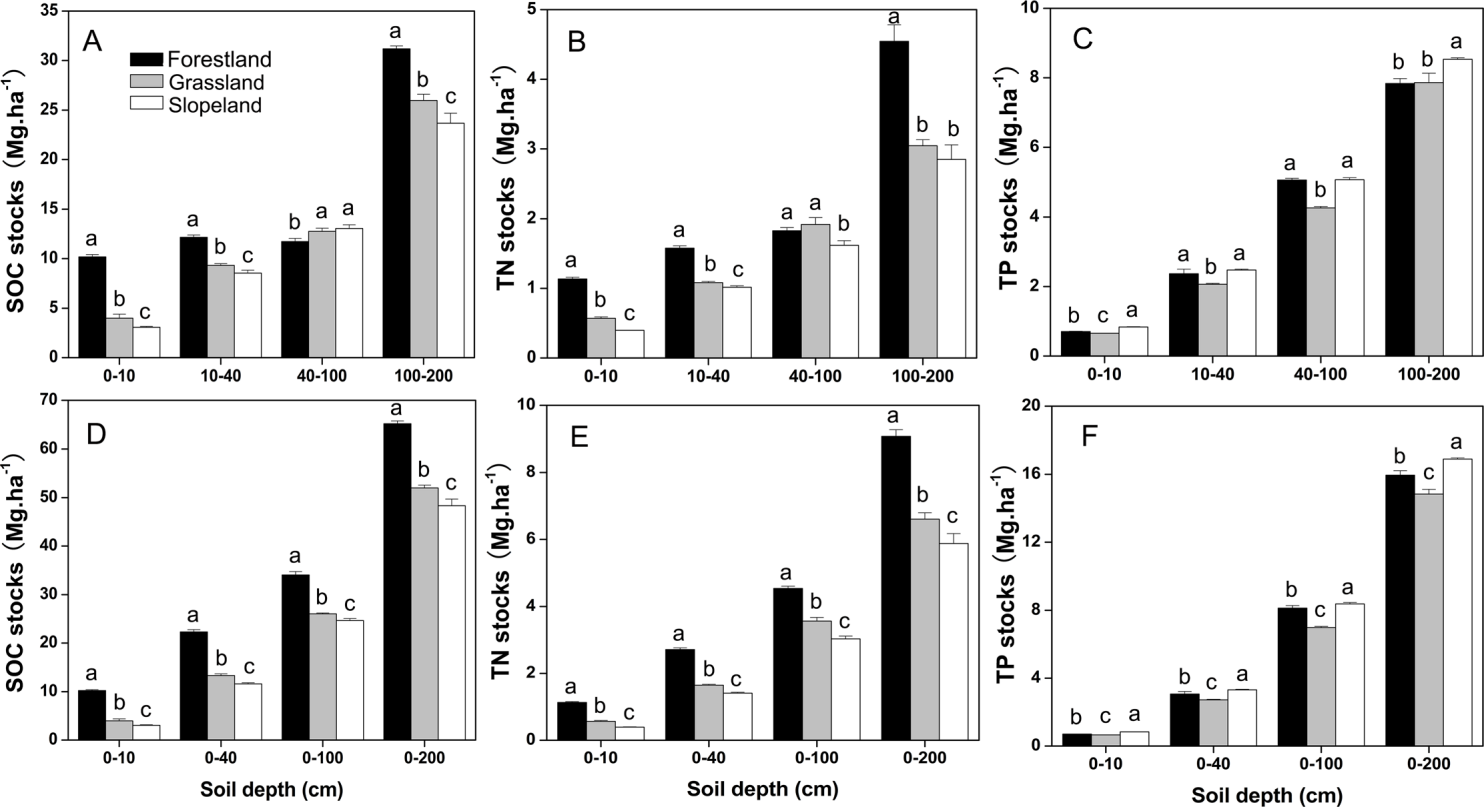
**Distribution of SOC stock (A), soil TN stock (B), TP stock (C), cumulative SOC stock (D), soil cumulative TN stock (E) and cumulative TP stock (F) at different soil depths.** The error bars are the standard errors. Different lowercase letters indicate significant differences at 0.05 (P < 0.05) levels among different land use types within the same soil layer.

Forestland and grassland had increased SOC and TN stocks, but decreased TP stocks (Fig 2). The SOC stocks of forestland and grassland were significantly higher than those in cropland at soil depths of 0–10 cm, 10–40 cm and 100–200 cm (P < 0.05); these values were significantly greater in forestland compared to grassland at these soil depths (P < 0.05) (Fig 2A). Compared with cropland, forestland had significantly increased TN stocks in all soil profiles (P < 0.05), and grassland also had increased TN stocks at all soil layers (Fig 2B). The TP stocks of forestland and cropland were significantly greater than those in grassland in soil depths of 0–10 cm, 10–40 cm and 40–100 cm, but forestland and grassland had significantly lower TP stocks than cropland at soil depths of 100–200 cm (Fig 2C). The cumulative SOC and TN stocks were significantly higher in forestland and grassland compared to cropland in all soil profiles; these values were significantly greater in forestland compared to grassland (P < 0.05) (Fig 2D and 2E). At soil depths of 0–200 cm in forestland and grassland, the cumulative SOC and TN stocks of forestland and grassland improved by 34.97% and 7.61%, respectively (SOC), and 54.54% and 12.47%, respectively (TN). The cumulative TP stock was significantly higher in cropland than in forestland and grassland, though this level was significantly greater in forestland compared to grassland (Fig 2F).

**Fig 3:**
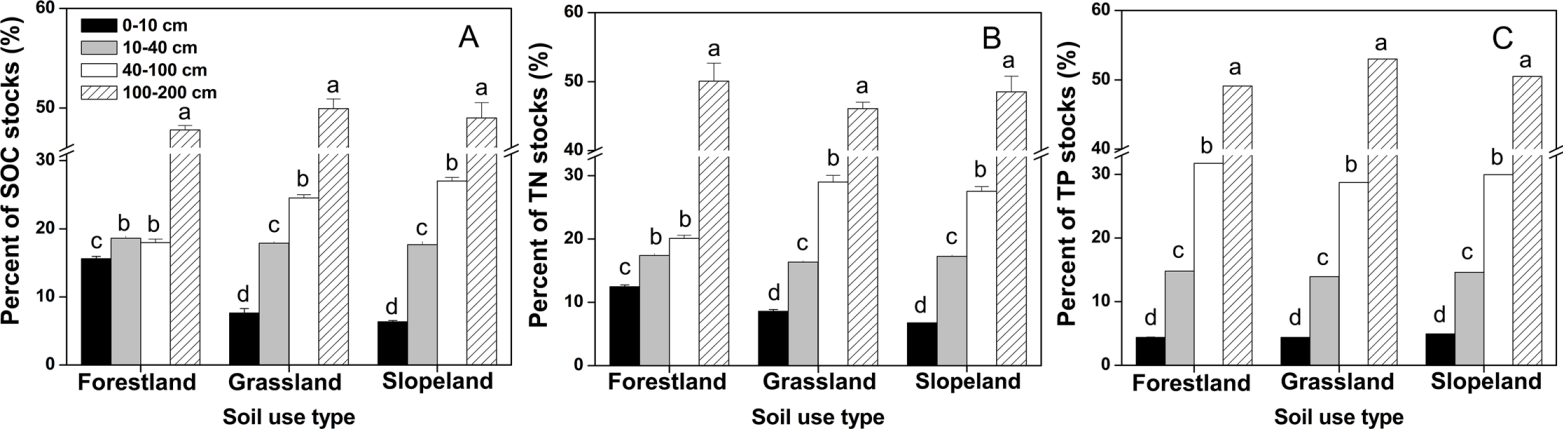
**Distribution ratios of the SOC (A), TN (B) and TP (C) stocks in soil at a depth of 0**–**200 cm.** The error bars are the standard errors. Different lowercase letters indicate significant differences at 0.05 (P < 0.05) levels among different soil depths within the same land use type.

The distributions of SOC, TN and TP stocks at different soil depths under all land use patterns were 0–10 cm < 10–40 cm < 40–100 cm < 100–200 cm except for the SOC stock of forestland at 40–100 cm (Fig 3). In all soil layers, the highest percentages of the SOC, TN and TP stocks were at a soil depth of 100–200 cm, accounting for 47.80%-49.93% (SOC), 46.08%-50.05% (TN) and 49.09%-52.98% (TP) (Fig 3); these percentages were significantly higher at this soil depth than at other soil depths (P < 0.05). The soil at 40–100 cm contained the second highest percentages of SOC, TN and TP stocks, with 17.98%-27.01%, 20.11%-28.98% and 28.69%-31.71%, respectively. The SOC, TN and TP stocks at soil depths of 0–10 cm were the lowest, accounting for 6.34%-15.61%, 6.74%-12.48% and 4.39%-4.95%, respectively. A two-way ANOVA indicated that land use patterns, soil depth and their interactions significantly affected the SOC, TN and TP stocks (Table 1).

### Changes in SOC, TN and TP stoichiometry

**Fig 4:**
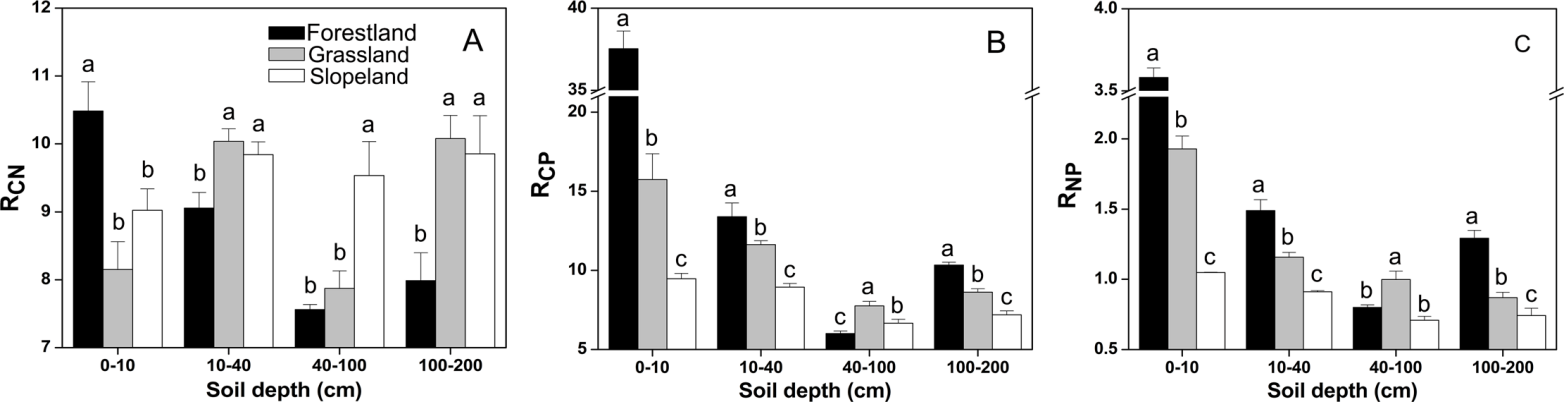
**Distribution of R**_**CN**_ **(A), R**_**CP**_ **(B) and R**_**NP**_ **(C) in soil at depths of 0**–**10 cm, 10–40 cm, 40–100 cm and 100–200 cm.** The error bars are the standard errors. Different lowercase letters indicate significant differences at 0.05 (P < 0.05) levels among the different land use types within the same soil layer.

The land use patterns had different effects on the soil R_CN_, R_CP_ and R_NP_ values. The soil C:N ratio of forestland was significantly higher than those of grassland and cropland at soil depths of 0–10 cm (P < 0.05), but lower than those of grassland and cropland at soil depths of 10–40 cm, 40–100 cm and 100–200 cm (Fig 4A). The C:N ratio did not significantly differ between grassland and cropland at soil depths of 0–10 cm, 10–40 cm and 100–200 cm (P > 0.05). The soil C:P and N:P ratios of forestland were significantly higher than those of grassland and cropland at soil depths of 0–10 cm, 10–40 cm and 100–200 cm; in these soil layers, the C:P and N:P ratios in grassland were significantly greater than those in cropland (P < 0.05) (Fig 4B and 4C). At soil depths of 40–100 cm, the C:P and N:P ratios of grassland were significantly higher than those of forestland and cropland (P < 0.05). Compared to grassland, the soil R_CP_ and R_NP_ values at different depths in forestland were significantly increased by 15.25%-138.41% and 28.73%-85.61%, respectively (P < 0.05) (Fig 4B and 4C). In all land use patterns, the soil C:N:P ratios gradually decreased at soil depths of 0–10 cm, 10–40 cm and 40–100 cm, then increased at a soil depth of 100–200 cm (Table 2). Two-way ANOVAs indicated that land use patterns, soil depth and their interactions significantly affected the soil C:N, C:P and N:P ratios (Table 1).

**Table 2:** Stoichiometry of soil C:N:P as affected by land use patterns and soil depth.

Soil depth (cm)	C:N:P as a function of land use patterns			
	Forestland	Grassland	Slopeland	Average
**0–10**	37.5: 3.6: 1	15.7: 1.9: 1	9.5: 1.0: 1	20.9: 2.2: 1
**10–40**	13.4: 1.5: 1	11.6: 1.2: 1	8.9: 0.9: 1	11.3: 1.2: 1
**40–100**	6.0: 0.8: 1	7.8: 1.0: 1	6.7: 0.7: 1	6.8: 0.8: 1
**100–200**	10.3: 1.3: 1	8.6: 0.9: 1	7.2: 0.7: 1	8.7: 1.0: 1
**Average**	11.0: 1.3: 1	9.3: 1.0: 1	7.5: 0.8: 1	9.3: 1.0: 1

### Relationship between stocks and stoichiometry of SOC, TN and TP

The correlations between stocks and stoichiometry of SOC, TN and TP among the different land use patterns were analyzed (Table 3). Positive correlations existed between the SOC and TN stocks at soil depths of 0–10 cm, 10–40 cm and 100–200 cm. The SOC and TN stocks also significantly correlated with the soil C:N, C:P and N:P ratios in each soil layer (P < 0.05). Meanwhile, significant correlations among the soil C:N, C:P and N:P ratios were discovered in most cases. However, no significant correlations were found between the TP stock and the SOC stock, the TN stock, the C:N ratio, the C:P ratio, or the N:P ratio.

**Table 3:** Pearson correlation coefﬁcients between soil C, N, P stocks and C:N:P ratios for different soil layers.

Soil depth (cm)		Cs	Ns	Ps	C:N	C:P
**0–10**	Ns	0.99 **	1			
	Ps	-0.37	-0.47	1		
	C:N	0.82 **	0.74 *	0.09	1	
	C:P	0.99 **	0.99 **	-0.46	0.78 *	1
	N:P	0.97 **	0.99 **	-0.58	0.67 *	0.99 **
**10–40**	Ns	0.99 **	1			
	Ps	0.08	0.16	1		
	C:N	-0.77 *	-0.87 **	-0.34	1	
	C:P	0.87 **	0.81 **	-0.41	-0.52	1
	N:P	0.94 **	0.91 **	-0.23	-0.71 *	0.97 **
**40–100**	Ns	-0.24	1			
	Ps	-0.30	-0.64	1		
	C:N	0.70 *	-0.86 **	0.33	1	
	C:P	0.68 *	0.41	-0.90 **	0.04	1
	N:P	0.06	0.90 **	-0.91 **	-0.63	0.75 *
**100–200**	Ns	0.94 **	1			
	Ps	-0.59	-0.49	1		
	C:N	-0.75 *	-0.93 **	0.33	1	
	C:P	0.97 **	0.89 **	-0.78 *	-0.69 *	1
	N:P	0.95 **	0.99 **	-0.62	-0.89 **	0.94 **

* Correlation significances were tested at P = 0.05 (P < 0.05) (1-tailed).

** Correlation significances were at P < 0.01 (2-tailed).

## Discussion

### Effects of land use pattern and soil depth on the SOC, TN, and TP contents and stocks

Our results showed that forestland and grassland enhanced the SOC and TN contents and stocks (Figs 1 and 2), which was consistent with a previous conclusion that converting cropland into perennial vegetation could increase the SOC and nutrient contents [43]. In our results, land use patterns, soil depth and their interactions all significantly affected the SOC, TN and TP contents and stocks (Table 1), indicating that land use and soil depth were the important factors influencing the soil nutrient distribution. The conclusion that forestland had the greatest SOC and TN contents and stocks, followed by grassland, and then by slope farmland (Figs 1 and 2) was consistent with Fu et al. [37]. Zhang et al. [40] showed similar result that SOC storages in the woodland, shrubland, grassland, orchard and dam cropland were signiﬁcantly higher than that in the sloped cropland. When soil depth was below 5 cm, orchards showed the maximum SOC contents [36], which may be attributed to the fact that farmers are accustomed to fertilizing orchards with organic manure and tillage operations, resulting in more residue and manure in subsurface soil [36]. The dam cropland exhibited higher C and N contents than sloped cropland due to the differences in crop types [40]. Fu et al. [37] reported that the TP content was greatest in cropland and lowest in grassland, which we also observed. One possible explanation is that TP seems to be preferentially saved in the soil of cropland [37].

Land use changes caused soil carbon and nutrient redistribution, and the revegetation of cropland increased soil carbon and nitrogen contents. It can be explained that cropland is withdrawn from cultivation after land use conversion, which corresponds to decreases in the decomposition and mineralization of soil organic matter. Another reason may be that perennial vegetation has a greater amount of residue input into the soil than cropland, thus resulting in the higher SOC and TN contents [34]. Compared with grassland, forestland holds larger amounts of C and N in the litter layer [44], resulting in higher SOC and TN contents in the topsoil. Moreover, forests have larger and deeper root systems than herbaceous plants, which therefore generate higher SOC and TN contents and stocks in the soil [45]. Furthermore, fine roots may contribute substantially to C and N changes in the deep soil [44].

Shi et al. [18] reported that afforestation on cropland increased SOC stores in each soil layer of mineral soils. Our study showed that forestland and grassland had higher SOC and TN stocks than cropland not only in the surface soil but also in the subsoil (Figs 1 and 2). The deep soil (40–200 cm) contained approximately 70%-80% of the cumulative SOC and TN stocks of the soil at depths from 0–200 cm (Fig 2), which was essentially consistent with previous studies [43, 46]. Carbon and nitrogen are largely stored in the deep soil mainly because organic matter protection in the subsoil spatially separates the soil organic matter, different particle fractions, microbes and extracellular enzyme activities related to the heterogeneity of C inputs [17]. In addition, SOC input into the subsoil is mostly influenced by vegetation roots, root exudates, and bioturbation, which may also play an effective role [43].

**Fig 5:**
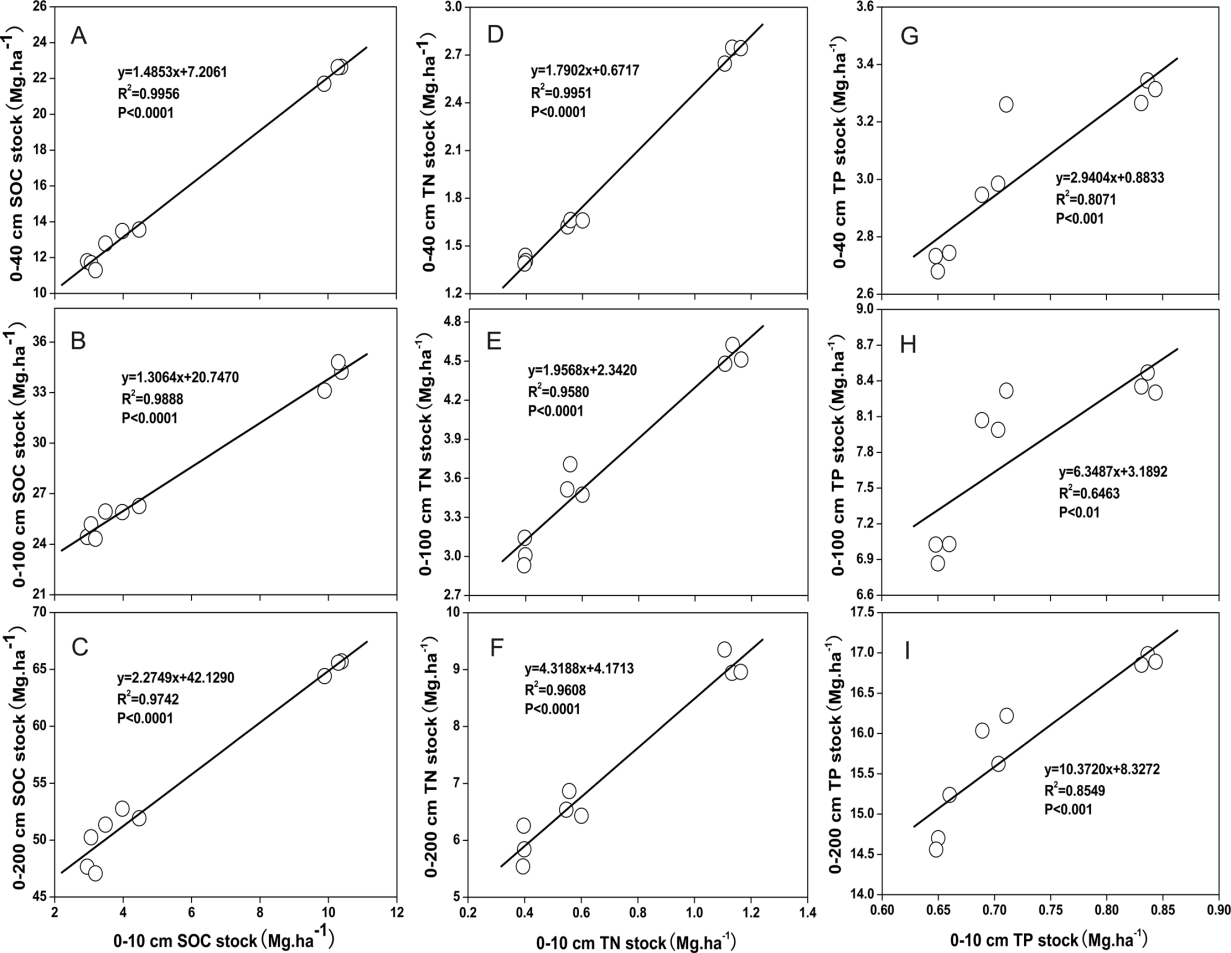
Relationships of SOC, TN and TP stocks between 0–10 cm and 0–40 cm, 0–100 cm, and 0–200 cm.

The relationship between the SOC, TN and TP stocks in the surface soil (0–10 cm) and in the subsoil (0–40 cm, 0–100 cm, and 0–200 cm) could be modeled using a linear regression function (y = ax + b) (Fig 5). Furthermore, the SOC, TN and TP stocks in the 0–10 cm layer accounted for 20.33%, 18.85% and 9.33% of the SOC, TN and TP stocks, respectively, in the 0–100 cm layer. Thus the C, N and P stocks in the deep soil layer could be calculated based on the data from the surface soil (0–10 cm). In our study, the average SOC stock was 5.74 Mg·ha^-1^ and 28.25 Mg·ha^-1^, respectively in the 0–10 cm and 0–100 cm layers, respectively. The average SOC stock in the entire Loess Plateau was calculated to be 11.4 Mg·ha^-1^ in the 0–20 cm layer and 45.5 Mg·ha^-1^ in the 0–100 cm layer, and in the Zhifanggou watershed, the mean SOC stocks in the 0–10 cm and 0–100 cm layers were 13.6 Mg·ha^-1^ and 41.6 Mg·ha^-1^, respectively [40]. Although topsoil layer was of a different thickness, the SOC stock in the 0–10 cm and 0–100 cm layers were far lower than in X (where X is whatever the 0–10 cm and 0–100 cm layers were being compared to). Therefore, additional attempts should be taken to improve the soil C and N conditions in the Wuliwan catchment.

### Effects of land use pattern and soil depth on C:N, C:P and N:P ratios

Generally, elements are present in relatively stable ratios in soil and living organisms [22]; these ratios are considered to determine the key characteristics of ecosystems [31]. A well-balanced global C:N:P ratio is 186:13:1 for surface soil (0–10 cm mineral soil) [13, 32]. In China, the general C:N:P ratios are 134:9:1 for 0–10 cm organic-rich soil and 60:5:1 for the entire soil depth (as deep as 250 cm) [33]. In our results, the average C:N:P ratio was 9.3:1:1 at the depth of 0–200 cm, and the highest C:N:P ratio was 37.5:3.5:1 at the depth of 0–10 cm (Table 2). These were far below the mean C:N:P ratios of China and Global. Based on a synthesis of observational data for the soils of China, Tian et al. [33] found well-constrained C:N (14.4), C:P (136) and N:P (9.3) molar ratios for the depth of 0–10 cm. Our study showed that long-term revegetation improved the soil C:P and N:P ratios. The largest soil C:N ratio was 10.49 (Fig 4A), and relatively consistent C:P and N:P ratio values of 37.53 and 3.58 (Fig 4B and 4C) were also found in the depth of 0–10 cm in forestland; these followed the normal distribution pattern of soil elemental ratios, with most C:N, C:P and N:P ratios in the ranges of 6–12, 24–48, and 3–6, respectively [33].

The low C:N ratio (< 25 on a mass basis) indicated that organic matter is decomposing faster than it is accumulating [22] and that net N mineralization is considered to happen [47]. C:N ratios lower than 10 imply that low levels of organic matter are being merged into the soil system [48, 49]. Our results showed that the land use patterns and soil depth had significant effects on the soil C:N ratio (Table 1). However, Wei et al. [47] suggested that the soil C:N ratio responds slowly to land use types and cannot reflect the changes of the SOC and TN statuses in the northern Loess Plateau. This may be due to differences in plant species. In our experiment, forestland had the lowest C:N ratio in the subsoil (10–40 cm, 40–100 cm) and deep soil (100–200 cm), which corresponded to the highest N:P ratio in almost all of the soil profiles (Fig 4). This may be ascribed mainly to the fact that *Rhizobium* associated with the root system of *Robinia pseudoacacia* fixed more nitrogen, leading to abundant levels of N in the subsoil and deep soil.

The soil C:P and N:P ratios had similar responses to land use patterns and soil depth (Fig 4B and 4C), and their distribution trends were consistent with those of the SOC and TN contents. As the soil TP changed only slightly with land uses and soil depth, the soil C:P and N:P ratios were determined by the SOC and TN contents. Soil in forestland and grassland have higher SOC and TN contents than the soil in cropland, resulting in increased C:P and N:P ratios. Notably, forestland had greater soil C:P and N:P ratios than grassland (Fig 4 and Table 2), which was consistent with the global soil nutrient ratios reported by Cleveland and Liptzin [32]. In addition, because the litter layer released more nutrients into the topsoil, this layer (0–10 cm) had greater soil C:P and N:P ratios than the subsoil or deeper soil. In our results, the soil C:P ratio ranged from 6.7 to 37.5, which implied a net mineralization of nutrients (< 200) [50].

**Fig 6:**
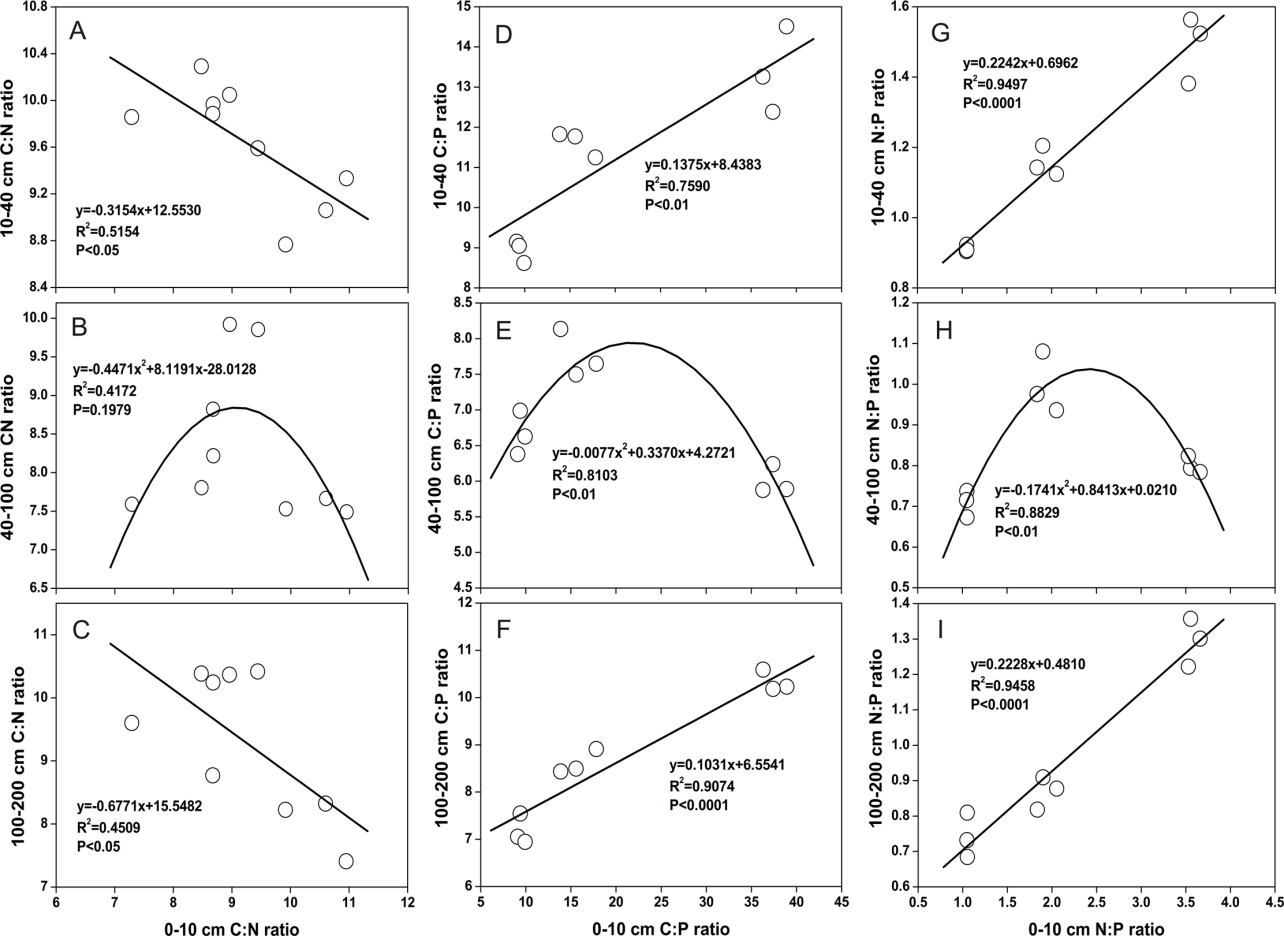
Relationship of C:N, C:P and N:P ratios between 0–10 cm and 10–40 cm, 40–100 cm, and 100–200 cm.

The relationship between the stoichiometry of SOC, TN and TP in the surface soil (0–10 cm) and in the subsoil (10–40 cm, 40–100 cm, and 100–200 cm) varied in different land use patterns and soil depths (Fig 6). This may have occurred for two reasons: first, there may have been differences in the absorption or release of C, N, and P from or to the soil by root systems [51, 52]; second, the release of N and P released into the soil by decomposing organic matter differs during litter decomposition processes [53].

In our study, the land use patterns, soil depth, and their interactions significantly influenced the soil C:N, C:P and N:P ratios (Table 1). Li et al. [54], Aponte et al. [55] and Zhao et al. [34] also concluded that various land use types display different soil C:N:P ratios. Li et al. [54] supposed that the differences in soil CNP stoichiometry may result from land management practices, vegetation types, and elevation. Zhao et al. [34] conjectured that only plant communities and vegetation affect soil nutrient stoichiometry. Fan et al. [56] demonstrated that the C:N, C:P and N:P ratios of leaves increased linearly with the soil C:N, C:P and N:P ratios and discovered that soil depth and the age of the plantation significantly influenced the stoichiometry of C, N and P in the soil [56]. In addition, our results showed that the soil SOC and TN stocks were significantly correlated with the soil C:N, C:P and N:P ratios in each layer (Table 3), indicating that the soil C and N stocks were also important factors influencing the soil C, N and P stoichiometry. Therefore, Zhang et al. [30] considered the soil C:N, C:P and N:P ratios to be highly complex, and more efforts should be made to understand the factors affecting the stoichiometry of C, N and P in the soil.

## Conclusions

Forestland and grassland enhanced the contents and stocks of SOC and TN, but lowered the soil TP content and stock. Forestland showed greater SOC, TN and TP contents and stocks than grassland. In the 40–200 cm soil layer, the SOC, TN and TP stocks accounted for approximately 70%-80% of the total SOC, TN and TP stocks in soils at depths of 0–200 cm, and at 100–200 cm, the SOC, TN and TP stocks accounted for nearly 50% of the total levels; these findings indicated the important role of deep soil. In addition, forestland had greater soil C:P, N:P and C:N:P ratios than either grassland or cropland. The land use patterns, soil depth and their interactions had significant effects on the contents, stocks and stoichiometry of SOC, TN and TP. Furthermore, the SOC and TN stocks were significantly correlated with the soil C:N, C:P and N:P ratios. Significant correlations also existed among the soil C:N, C:P and N:P ratios in most cases. These results indicated that revegetation improved the soil nutrient status, and afforestation is a more effective means of restoring the soil of degraded cropland. Deep soil is significantly important in assessing the soil C, N and P pools and stoichiometry. In the present study, we only chose representative land use patterns, but a future study might include more land use types and age sequences.
